# Community-based tuberculosis contact management: Caregiver experience and factors promoting adherence to preventive therapy

**DOI:** 10.1371/journal.pgph.0001920

**Published:** 2023-07-14

**Authors:** Micaela Sandoval, Godwin Mtetwa, Tara Devezin, Debrah Vambe, Joyce Sibanda, Gloria S. Dube, Thandeka Dlamini-Simelane, Bhekumusa Lukhele, Anna M. Mandalakas, Alexander Kay

**Affiliations:** 1 The Global Tuberculosis Program, Texas Children’s Hospital, Baylor College of Medicine, Houston, Texas, United States of America; 2 Epidemiology, Human Genetics & Environmental Sciences, UTHealth School of Public Health, Houston, Texas, United States of America; 3 Baylor College of Medicine Children’s Foundation-Eswatini, Mbabane, Eswatini; 4 Eswatini National Tuberculosis Control Program, Manzini, Eswatini; 5 Centre for the Advancement of Scholarship, University of Pretoria, Pretoria, South Africa; 6 Health Policy & Organization, School of Public Health, University of Alabama at Birmingham, Birmingham, Alabama, United States of America; 7 Clinical Infectious Disease Group, German Center for Infectious Research (DZIF), Clinical TB Unit, Research Center Borstel, Borstel, Germany; University of Embu, KENYA

## Abstract

Delivery of tuberculosis preventive therapy (TPT) for children with household exposure to tuberculosis is a globally supported intervention to reduce the impact of tuberculosis disease (TB) in vulnerable children; however, it is sub-optimally implemented in most high-burden settings. As part of a community-based household contact management program, we evaluated predictors of adherence to community based TPT in children and performed qualitative assessments of caregiver experiences. The Vikela Ekhaya (Protect the Home) project was a community-based household contact management program implemented between 2019 and 2020 in the Hhohho Region of Eswatini. At home visits, contact management teams screened children for TB, initiated TPT when indicated and performed follow-up assessments reviewing TPT adherence. TPT non-adherence was defined as either two self-reported missed doses or a pill count indicating at least two missed doses, and risk factors were evaluated using multivariate clustered Cox regression models. Semi-structured interviews were performed with caregivers to assess acceptability of home visits for TPT administration. In total, 278 children under 15 years initiated TPT and 96% completed TPT through the Vikela Ekhaya project. Risk factors for TPT non-adherence among children initiating 3HR included low family income (adjusted hazard ratio (aHR) 2.3, 95%CI 1.2–4.4), female gender of the child (aHR 2.5, 95% CI 1.4–5.0) and an urban living environment (aHR 3.1, 95%CI 1.6–6.0). Children with non-adherence at the first follow-up visit were 9.1 fold more likely not to complete therapy. Caregivers indicated an appreciation for community services, citing increased comfort, reduced cost, and support from community members. Our results are supportive of recent World Health Organization (WHO) recommendations for decentralization of TB preventive services. Here, we identify populations that may benefit from additional support to promote TPT adherence, but overall demonstrate a clear preference for and excellent outcomes with community based TPT delivery.

## Introduction

Tuberculosis preventive therapy (TPT) is a cornerstone of tuberculosis (TB) prevention strategies in children. For over 60 years, TPT has been shown to reduce the risk of developing TB disease in children following exposure to a person with infectious TB [1–3]. This benefit is most well established in children under five years of age following TB exposure [3], because of the higher rate of progression to TB disease and the greater risk of severe disease in this population [4–6]. Preventive therapy in this population is universally recommended following exposure to bacteriologically confirmed TB patients [7]; however, there are differences in implementation. In low-resource, high burden settings, immunological tests of infection are not cost-effective [8] or sufficiently predictive of progression to TB disease in this population [9]. Additionally, chest radiography is not considered necessary to exclude TB disease based on the high negative predictive value of symptom assessments alone [10]. Therefore, recommendations suggest the use of a symptom based approach for TPT implementation to eliminate barriers arising from use of chest radiography and tests of infection to define eligibility for TPT [7]. Despite this approach, the number of children under 5 years receiving TPT has not increased annually since 2019 [11], and the total number of TPT courses accepted by this population only reached 40% of the cumulative targets for 2018–2022 set by the UN high-level meeting on TB [11]. In spite of evidence-based initiatives that promote TPT implementation, a complex network of barriers preventing effective implementation remain including the duration of TPT and access to TPT [12].

If adopted by national TB programs, child-friendly, dispersible, fixed-dose drug combinations present an opportunity for more acceptable and effective TPT [13]. Since 2018, the WHO has strongly recommended 3HR (three months of daily, fixed-dose, isoniazid and rifampicin) for the preventive treatment of children under 15 years in high TB incidence countries. 3HR is effective [14] and can be delivered with child-friendly formulations, access to isoniazid and rifampicin was already established, and health care providers were familiar with the medication [7]. Further, data from multiple settings suggests that shorter rifamycin based TPT regimens such as 3HR result in higher rates of completion in children [15–17].

Despite these advances in drug technology, reductions in treatment duration, and symptom based eligibility criteria, numerous well-recognized barriers to child contact management persist, including poor access to care, inadequate health infrastructure, limited knowledge coupled with unfavorable attitudes and misperceptions in patients and health care workers, and stigma [12]. Community-based contact management addresses many of these structural barriers [18], and has been found to be feasible and acceptable to patients and health care workers [19]. Increasingly, the WHO emphasizes the need for decentralized models of care [20] informed by emerging data suggesting that community-based preventive services can dramatically increase uptake [21].

Developed to address these barriers, Vikela Ekhaya (Protect the Home) was a novel, differentiated, community-based contact management program implemented in the Hhohho Region of Eswatini from April 2019 to March 2020 [22]. Embracing community management, this project provided 3HR for children following household exposure to tuberculosis or six months of isoniazid in a limited proportion of children ineligible for 3HR. Consistent with data on the benefits of this approach, this implementation project demonstrated TPT uptake and completion of greater than 93% [22]. To guide programmatic decentralization of tuberculosis contact management in high-burden settings, we now describe the predictors and measures of adherence among child contacts, tolerability of 3HR as a TPT regimen, and acceptability of the intervention to participating families.

## Methods

### Vikela Ekhaya program

Vikela Ekhaya was implemented as a TB Reach project by the Baylor Children’s Foundation-Eswatini in collaboration with the Eswatini National Tuberculosis Control Program and Baylor College of Medicine. The Vikela Ekhaya program was conducted in the Hhohho region of Eswatini and targeted child contacts of adult TB cases. Implementation science was integrated into the program using mixed methods, inclusive of semi-structured interview tools for qualitative assessments and measurements of participant characteristics and adherence for quantitative analysis. A full description of project activities has been published previously [22]. In brief, patients with TB were offered the choice of community or facility-based contact management. For those patients who chose community management, mobile contact management teams visited their homes to evaluate household contacts for TB symptoms, or other signs of illness, to initiate TPT if eligible, and to follow patients until completion of TPT. This project provided three months of fixed dose rifampicin and isoniazid (3HR) for children without HIV or 6H for children living with HIV; 6H was also offered when there were concerns regarding potential drug-drug interactions to rifampicin or there were stock outs of HR. Follow-up visits were made every four to six weeks to assess secondary measures of adherence including pill counts, missed doses, and adverse events, and to document TPT outcomes. Similar activities were performed in the facility management visits.

Contacts were considered to have successfully completed therapy if they reported that they finished at least 80% of the required doses within four months (3HR) or nine months (6H), in accordance with WHO guidelines [23]. Clinical teams instructed caregivers to discontinue TPT if they suspected or diagnosed TB disease or observed a severe adverse reaction and documented participant-initiated discontinuation. Dates were captured for completion and discontinuation of TPT, as well as the reason for discontinuation.

### Programmatic assessment

In addition to pill counts collected by study nurses, caregivers recorded TPT doses using a tracking card. At each visit, team members asked caregivers to rate the difficulty of administering TPT, the acceptability of TPT, and the time required to administer TPT using a Likert scale; caregivers of young children were additionally asked if the child had vomited the medicine or if the child was experiencing any side effects. All child contacts receiving TPT were followed to either treatment discontinuation, treatment completion, treatment time-out, or loss to follow-up.

Additional information about caregivers’ experiences with TB, community-based contact management, and TPT administration was obtained from in-depth semi-structured interviews conducted at the conclusion of contact management. Families were selected through purposive random sampling, from all households who had chosen to participate in community-based contact management and invited to participate in a semi-structured interview in their homes. Caregivers were interviewed until data saturation was reached (N = 14).

Audio-recorded interviews were conducted in siSwati and transcribed in siSwati by a trained social worker. Audio recordings were then translated into English by trained translators. Major themes, including experience of TPT administration and perceptions of community-based contact management, were extracted from translated transcriptions of audio recordings through textual review and sorting using open-coding [24].

### Statistical analysis

For this analysis, non-completion and non-adherence were measured separately. The primary measure of non-adherence was operationally defined as the first recorded instance of two or more missed doses to avoid misclassification of contacts who took their daily dose at or after their follow-up visits. Related measures of adherence, including pill counts, tracking cards, and self-reported missed doses, were reviewed for consistency, and agreement between pairs of measures was determined using Cohen’s kappa coefficient. For time-to-event analysis, time 0 represents the date of TPT initiation. Data was right censored at treatment completion cutoff dates (four months for 3HR and nine months for 6H).

Demographic and clinical data was reported as frequencies and proportions for categorical variables and as median and interquartile range (IQR) for continuous variables. Clustered error estimations were used to compensate for correlation within households. Complete household characteristics were only available for families who chose community-based contact management; therefore, families who chose to receive facility-based management were excluded from regression analyses. Similarly, models included only children under 5 because of substantial variations in the etiology of non-adherence in children under 5 compared with older children [25]. Univariate and multivariable clustered Cox regression analyses were performed to determine the characteristics associated with the hazard of 3HR non-adherence. Variable selection for the multivariable hazard model was based on the results of univariate analysis and clinical relevance. All analyses were performed on Stata version 16.0 (StataCorp LLC, College Station, TX, USA). A p-value of <0.05 was considered statistically significant.

### Ethical considerations

All clinical investigation supporting the reporting of these findings was conducted according to the principles expressed in the Declaration of Helsinki. All participants gave verbal consent for participation in the Vikela Ekhaya program. Verbal consent was obtained by project staff with a script and documented in the project database, this approach was approved by all ethics boards. Approval for the project was obtained from all necessary ethical bodies including the Baylor College of Medicine Children’s Foundation Eswatini (00013367), the Eswatini National Health Research Review Board (00025854/SHR326/2020), and the Baylor College of Medicine Institutional Review Board (H-35028), Houston, Texas, USA.

## Results

**Fig 1:**
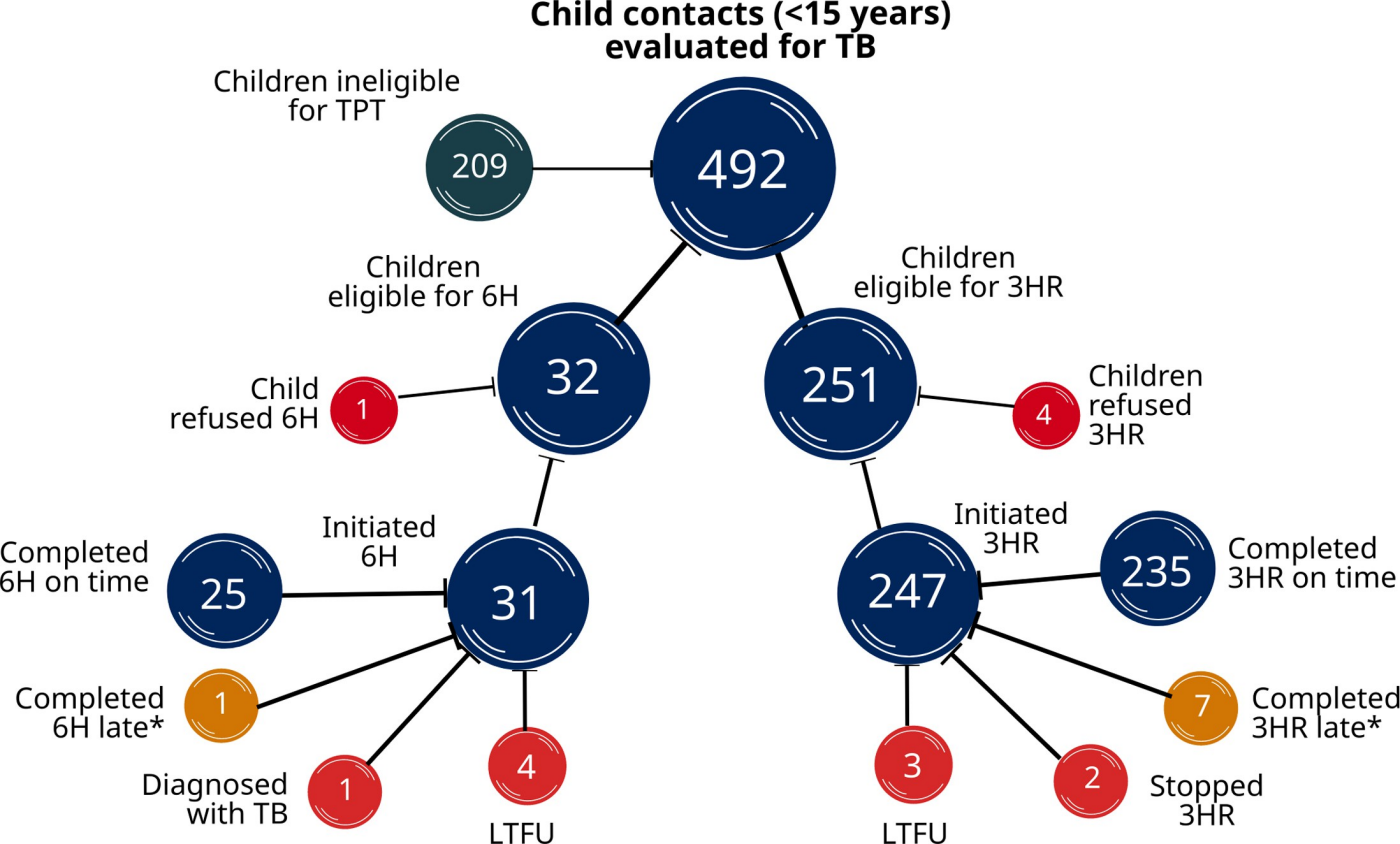
Visual representation of the flow of child contacts 0 to 14 years of age evaluated for TB following household exposure. The ineligible population is largely comprised of children 5–14, in whom TPT was not recommended by National Guidelines unless requested by the household.

In total, 251 children under 15 years of age were eligible to initiate 3HR, while 32 children were eligible for 6H; caregivers refused TPT for five children in total (one (4%) 6H-eligible and four (2%) 3HR- eligible). Of the total 278 children who initiated TPT, 271 (97%) did so in the community. Among children initiating, 235 (95%) completed 3HR within four months, and 25 (81%) completed 6H within nine months, in compliance with WHO TPT guidelines (Fig 1). Three children discontinued TPT early for the following reasons: side effects, caregiver request, and progression to TB disease in a child living with HIV. Fifteen children who initiated TPT were lost to follow-up (n = 7) or completed their assigned regimens after the extended treatment completion deadline (n = 8), according to WHO guidelines (10).

In children initiating TPT (Table 1), the vast majority were under five; 91.9% in children initiated on 3HR and 84% in children initiated on 6H, due to the higher proportion of children living with HIV in this group. Notably, 94% of children under five who initiated 3HR had known HIV status at time of screening. Contacts who initiated TPT came from primarily rural households with four or more members who were screened for TB and had a monthly household income of greater than 70 US dollars. Despite most families being within one hour of travel time to the clinic, 78 (97%) elected household TPT management.

**Table 1:** Characteristics of all household contacts zero to 14 years initiating TPT by regimen.

	Initiated 3HR	Initiated 6H
	N = 247	N = 31
Gender		
Female	123 (49.8%)	14 (45.2%)
Male	124 (50.2%)	17 (54.8%)
Age		
Child (5–14 years)	20 (8.1%)	5 (16.1%)
Infant (0–4 years)	227 (91.9%)	26 (83.9%)
Knowledge of HIV Status		
Known	231 (93.5%)	31 (100.0%)
Unknown	16 (6.5%)	0 (0.0%)
HIV status		
Reactive	0 (0.0%)	9 (29.0%)
Non-reactive	247 (100.0%)	22 (71.0%)
Number of contacts per household		
1–3 Contacts	63 (25.5%)	11 (35.5%)
4–6 Contacts	97 (39.3%)	15 (48.4%)
7–9 Contacts	50 (20.2%)	4 (12.9%)
10+ Contacts	37 (15.0%)	1 (3.2%)
Travel time to facility		
0 to 30 minutes	152 (61.5%)	24 (77.4%)
30 minutes to 1 hour	72 (29.1%)	6 (19.4%)
1 hour+	15 (6.1%)	0 (0.0%)
Missing	8 (3.2%)	1 (3.2%)
Monthly household income (USD)		
0–35	13 (5.3%)	0 (0.0%)
36–70	33 (13.4%)	3 (9.7%)
> 70	193 (78.1%)	27 (87.1%)
Missing	8 (3.2%)	1 (3.2%)
Household setting		
Rural	209 (84.6%)	24 (77.4%)
Urban	29 (11.8%)	6 (19.4%)
Missing	9 (3.6%)	1 (3.2%)
TPT management		
Community	242 (98.0%)	29 (93.5%)
Facility	5 (2.0%)	2 (6.5%)
TPT Outcome		
Completed	235 (95.1%)	25 (80.6%)
Family or Patient Discontinued	1 (0.4%)	0 (0.0%)
LTFU	3 (1.2%)	4 (12.9%)
Discontinued due to side effect	1 (0.4%)	0 (0.0%)
Timed out	7 (2.8%)	1 (3.2%)
TPT Failure	0 (0.0%)	1 (3.2%)

Within this cohort of households receiving almost exclusively community-based care, tracking cards were not an effective or reliable method of measuring adherence; most caregivers either did not use them at all or used them incorrectly, i.e., tracking pill number and calendar day interchangeably. However, patient-reported missed doses and clinician-recorded pill counts were both consistently collected and recorded, and agreement between the two measures was 83% using Cohen’s kappa. Based on these analyses, a composite measure of non-adherence was created from the self-reported and pill count measures: the non-adherence event was defined as the first instance of missing two or more daily doses within the last four to six-week interval, either by self-report or pill count.

Seven children who chose to receive facility based TPT management and six children for whom adherence data was not available were excluded from adherence analyses. Of the 217 children under five who received community based TPT with 3HR and had adherence data available, 58 (27%) were non-adherent at some point (Table 2). Characteristics of the sub-cohort of children under five are described in S1 Table. In multivariable Cox regression models, the adjusted hazard ratio for female gender (aHR 2.5, 95% confidence interval (95%CI) 1.4–5.0), low household income (aHR 2.3, 95% CI 1.2–4.4), and urban setting (aHR 3.1, 95% CI 1.6–6.0) emerged as risk factors for non-adherence to TPT. Due to the small number of children receiving 6H, this population was excluded from the time to event analysis.

**Table 2:** Clustered cox regression: Risk factors for non-adherence to 3HR TPT regimen among child contacts under five.

Event: first instance of non-adherence to 3HR*, n = 58	Univariable Analysis		Multivariable Analysis	
	N = 217		N = 217	
	HR (95% CI)	p-value	aHR (95% CI)	p-value
Gender				
Female	2.5 (1.4–5.0)	<0.001	2.5 (1.4–5.0)	0.001
Male	REF		REF	
Knowledge of HIV Status				
Known	REF			
Unknown	1.3 (0.5–3.0)	0.0575	—	
Travel time to facility				
0 to 30 minutes	REF			
30 minutes to 1 hour	1.1 (0.5–2.2)	0.805	—	
1 hour+	0.6 (0.1–4.9)	0.664	—	
Monthly household income (USD)				
0 to 70	2.3 (1.2–4.6)	0.014	2.3 (1.2–4.4)	0.008
> 70	REF		REF	
Household setting				
Rural	REF		REF	
Urban	3.2 (1.6–6.2)	0.001	3.1 (1.6–6.0)	0.001

*TPT*: TB preventive therapy. *HR*: Hazard Ratio. *CI*: Confidence Interval. *FU*:Follow-up. *LTFU*: Loss to follow-up.

* TPT non-adherence event defined as first instance of two or more missed daily doses, identified by self-report or nurse-conducted pill counts. *Note*: *Five children who chose facility based TPT management and five children who chose for whom adherence data was not available are excluded from this model*.

**Fig 2:**
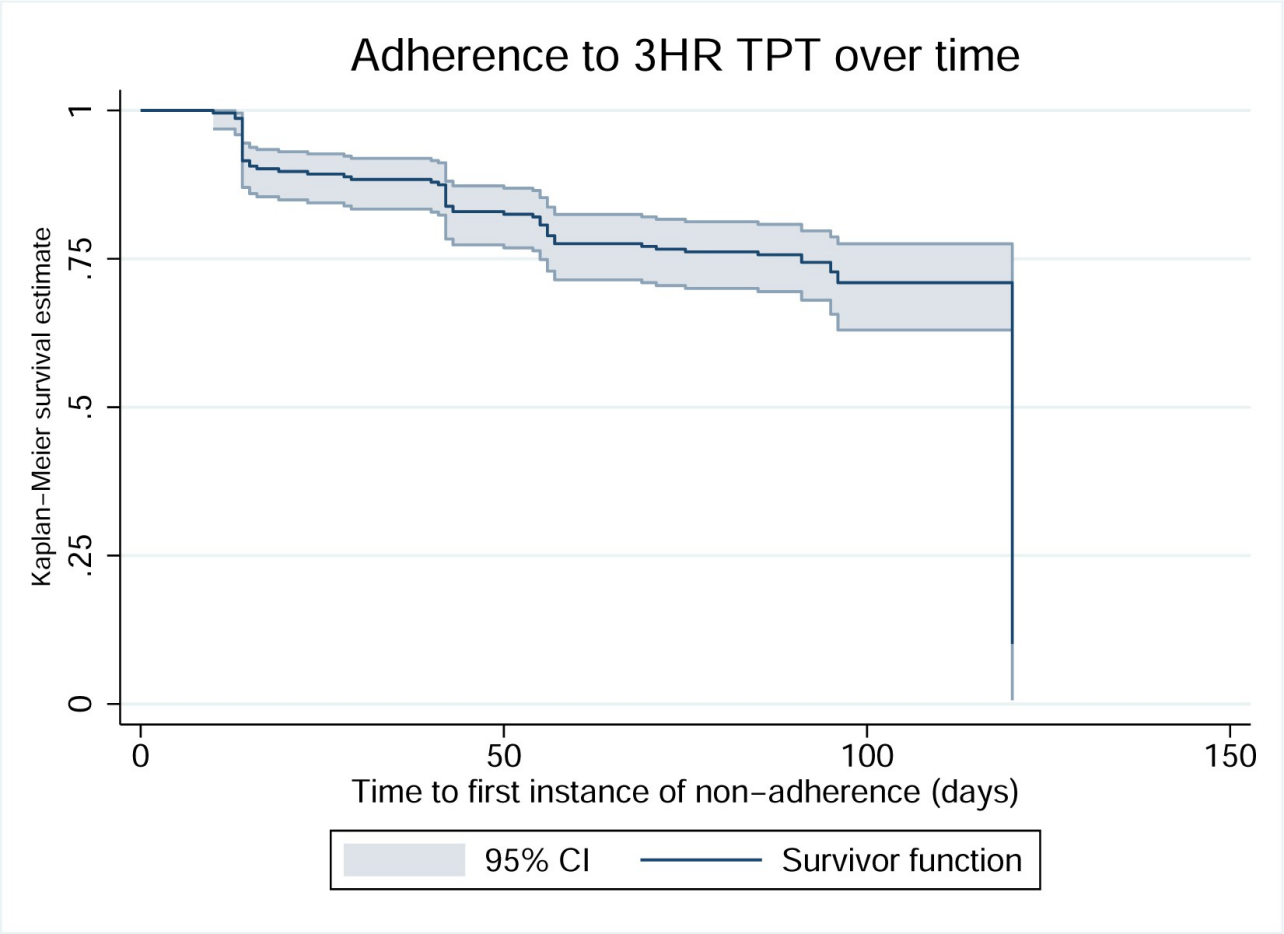
Kaplan Meier curve demonstrating the time to the first instance of non-adherence. Non-adherence was defined as 2 or more missed doses within the prior four-to-six-week interval as measured by self-report or pill count.

Within the population of 18 children who did not complete therapy on time, eight (44%) had missed at least two daily doses between initiating TPT and the first follow-up visit (approximately 30 days). This is demonstrated visually in a Kaplan Meier curve (Fig 2). Overall, children receiving 6H were more likely to not to complete TPT (odds ratio (OR) 5.2, 95% CI 1.6–17.1). A univariable clustered logistic regression analysis was conducted to determine whether the composite non-adherence variable was associated with non-completion among all contacts receiving TPT (Table 3). Children who had a non-adherent outcome at the first follow-up visit were more likely not to complete TPT (OR 9.1, 95% CI 2.7–30.6). There was not an impact on the odds of completion of TPT in children with a non-adherent event at the next follow-up visit (OR 2.6, 95% CI 0.5–13.9).

**Table 3:** Outcomes of children zero to 14 years by TPT regimen and composite adherence measure.

Outcome: TPT non-completion*, n = 16		Univariable analysis	
		N = 265	
		OR *(95%CI)*	*P-value*
TPT Regimen	3HR	REF	
	6H	5.2 (*1*.*6–17*.*1*)	*0*.*006*
Adherence**	Adherent beyond 2nd FU visit	REF	
	Non-adherent at 1st FU visit	9.1 (2.7–*30.6*)	*<0*.*001*
	Non-adherent at 2nd FU visit	2.6 (*0*.*5–13*.*9*)	*0*.*251*

TPT: TB preventive therapy. OR: Odds Ratio. CI: Confidence Interval. FU:Follow-up.

* TPT non-completion defined as stopping TPT for any reason, leaving monitored TPT management (LTFU), or failing to complete 90% of the assigned regimen within 133% of the expected time, according to WHO definitions.

**First recorded missed dose, by self-reported missed dose or pill counts. Note: Seven children who chose to receive facility based TPT management and six children for whom adherence data was not available are excluded from this model

Of the 271 caregivers who completed TPT follow up visits, 34 (13%) gave responses other than *most acceptable (number 1)* on the acceptability of TPT Likert at some point during the therapy process. Further, only four (1%) caregivers gave responses other than 1: *least difficult* at some point using the difficulty of TPT Likert scale. Of the fourteen families who participated in semi-structured interviews, thirteen had accepted TPT for their eligible children and successfully completed their treatment; one family refused TPT for their child and declined a referral to the TB clinic for further evaluation. All families who had accepted TPT were administering 3HR. Caregivers who described feeling uncertain about TPT were concerned about giving medication to children without any TB symptoms (Table 4). When asked whether they would prefer to give two medicines combined in one dissolvable pill for three months (3HR) or one tablet daily for six months (6H), caregivers gave mixed responses. Caregivers who preferred a six-month regimen either felt they would receive more care and attention during a six-month treatment or that a longer treatment would be more effective. In contrast, the caregivers who chose the three-month regimen said it would be easier to administer a shorter regimen to young children. Caregivers appreciated the motivation and support the nurses provided during TPT administration, as well as the child-friendly educational materials provided by the program. Female caregivers highlighted concerns about TPT from male-family members who may not have been present during the community visit, and the challenges they posed for them with respect to TPT administration. Caregivers universally appreciated receiving services at home due to convenience, comfort, community support and cost savings.

**Table 4:** Caregivers’ experience of providing community-based TB preventive therapy for their children.

Perceptions of TPT	"When she started, I wasn’t happy because she didn’t have symptoms; I was scared of possible side effects." (*HIV-/TB+ woman; gave 3HR to one child*)
	"The children were given TPT to prevent getting TB, that made me happy." (*HIV- woman; gave 3HR to one child*)
	"The children’s fathers do not understand, (they) think they will get TB if they take TPT." (*HIV- woman; gave 3HR to one child*)
	“I never gave my son the medication… my husband kept saying our son does not have TB” (*Woman; declined TPT*)
Experience of administering TPT	"It was very hard, pills are bitter." (*HIV- woman; gave 3HR to one child*)
	"It is very difficult, they don’t want them, I really have to be patient with them" (*HIV+ TB pos woman; gave 3HR to two children*)
	"It was easy, he liked taking TPT because I also take pills…and we would take them together" (*HIV pos*, *TB pos woman; gave 3HR to two children*)
	“(My child) does not have a problem with taking medication. As a matter of fact, she would remind you that it is time to take medication” (*HIV- woman; gave 3HR to one child*)
Preferences for TPT regimen	"You are given special attention when you take pills for longer … 6 months is better, I would know for sure that TB is gone." (*Woman; gave 3HR to one child*)
	"I will feel better to take for 6 months, that way I will know I am well" (*HIV neg*, *TB pos woman; gave 3HR to one child*)
	"3 months is better, the period is very short, its better" (*HIV pos*, *woman; gave 3HR to four children*)
	"3 months, you don’t want to give this kind of medication for long because children get tired of taking meds" (*HIV+ woman; gave 3HR to one child*)
Experience of community-based contact management	"I’m happy because you are here to help. It’s always difficult to go to the clinic because it’s expensive. " (*HIV- woman; gave 3HR to one child*)
	"Household care is much better, I wasn’t going to take my children, all 4 of them to the clinic… I would have defaulted.” (*HIV+ woman; gave 3HR to four children*)
	"Yes, (neighbors) were very supportive and I am happy about that. They did not gossip about us.” (*Woman; gave 3HR to one child*)
	"I am also able to ask a lot of questions … I am in my comfort zone. The nurse has time for us here, unlike in the clinics." (*HIV-/TB+ woman; gave 3HR to one child*)

## Discussion

The Vikela Ekhaya program demonstrated the feasibility of a differentiated care model allowing household contacts of TB cases to receive TPT and adherence support at their homes in an area with high TB and HIV burden and few public health resources. Small mobile contact management teams were able to efficiently follow families located across the Hhohho Region, which covers approximately 25% of the area and 30% of the population of Eswatini. The mobile teams answered questions, provided encouragement, treated other minor ailments unrelated to TB, and monitored recipients for side effects and adverse events from TPT eligibility screening through initiation and completion.

Overall, Vikela Ekhaya provided TPT to 208 children under five years in Hhohho in the year 2019, of whom 191 (92%) completed their regimens on time. This high rate of completion aligns well with the few other published reports of community based TPT [26, 27]. National notification data indicates only 307 child contacts under five years initiated TPT across the entire country of Eswatini over that same time-period. Furthermore, in 2015, our team documented a 7% TPT initiation rate among Swazi child contacts receiving routine facility-based contact management [28]. Although the decision to initiate TPT is complex and often dependent upon the caretaker’s knowledge, attitude and behavior, this collective data suggests that community management can have a substantial impact on TPT uptake [29].

Children receiving community based TPT delivery were most adherent in rural households and in families with a monthly household income greater than 70 USD. As of 2016, approximately 36% of the population in Eswatini falls below this income level according to World Bank estimates [30]. The fact that a greater proportion of households in our study had an income above this threshold likely speaks to the combined income of multiple adults within the home. Overall, these findings suggest that within a community based differentiated care model, even after eliminating most financial barriers to care, populations with fewer financial resources and those in more urban settings, who could have less extensive family networks and less privacy in shared accommodation common in urban areas [31, 32], may need additional support to maintain good adherence and complete TPT. This data aligns well with existing research demonstrating a clear link between child poverty and general health outcomes in low- and middle-income countries; elimination of costs may not be enough to level the playing field for children in poverty without additional unrestricted cash transfers [33, 34]. The factors identified were also similar to those identified in another study among caregivers administering TPT for children living with HIV, where financial insecurity also featured prominently [25]. However, in contrast to children living with HIV in whom stigma reduced adherence [25, 35], within the Vikela Ekhaya cohort, of predominantly HIV negative children, stigma did not appear to be an issue for families receiving community care and was not identified as a barrier to adherence.

Among children under five years who received 3HR, girls were also at higher risk of non-adherence than boys. This finding requires further qualitative and quantitative investigation to determine if patriarchal social norms are indeed a barrier to equitable TPT delivery, and, if so, to develop appropriate behavioral health solutions. We also found through interviews that female caregivers were sometimes unable to independently make decisions about TPT administration within their family structures, and only after discussions at the household between the female caregiver or the health care team and the familial patriarchs, did TPT adherence improve or commence. This again speaks to the importance of community engagement, where health care workers are more likely to encounter the key decision makers within the family structure, and these cultural norms can be adhered to and more effectively addressed [36]. While respecting cultural norms, community based care also increases access for women and girls and can provide support for female caregiver perspectives by engaging with the entire household [37].

Early non-adherence identified by detailed and restrictive assessments, such as those used in this study, was also associated with a decreased likelihood of TPT completion. This suggests that detailed screening for any missed doses by self-report may also provide an intervention opportunity for additional counseling and motivational interviewing on TPT completion. This type of intervention requires that health care workers are well trained on indications for TPT and the benefits of TPT to interact with family members with reservations effectively [38]. In interviews, families reported that their positive interactions with the health care teams at their homes were one of the key drivers for TPT completion [39]. The largest drop in adherence was observed at the first longitudinal follow-up visit, perhaps prior to families establishing trust with the health care team. Regardless of the root-cause, the initial period of TPT represents a key timepoint for enhanced adherence discussions to allow for completion within appropriate timeframe.

Overall, both TPT regimens were well tolerated. Only eight (3%) children experienced any minor side effects or regurgitation, according to their caregivers. Caregivers interviewed in more detail about their experiences with TPT administration indicated regurgitation was incidental, and difficulty administering the medications was not a major factor in their decision to continue treatment. Difficulty administering the medications was rare but was still reported in semi-structured interviews with caregivers giving the HR fixed dose combination (FDCs). This general approval of the FDCs, but with significant variability in personal experience is consistent with existing data [40]. Overall, child-friendly formulations are likely to make a difference for many families, but probably will not be the primary driver of TPT uptake and completion. Duration of therapy was important to caregivers in this study and the high-level of completion spoke to the benefits of 3HR. Although some families preferred a longer TPT regimen in this study for added assurance that the medication was working, data is clear from this study and others that longer TPT durations are associated with reductions in adherence [14, 15, 17, 41].

Although this investigation was strengthened by the availability of longitudinal data points, multiple robust measures of adherence, and in-depth participant interviews, analyses were limited to operational research and post-hoc investigations of this programmatic intervention. For example, because families impacted by TB were offered free choice of facility-based or community-based contact management and TPT delivery, and the assignment of 6H versus 3HR regimens was based on national treatment algorithms, we could not balance the cohort across exposure subpopulations. Additionally, because household characteristics data was only available for households who had been visited by the mobile contact management teams at some point, we excluded the few contacts who exclusively received facility-based care in regression analyses. This limited our power to assess our interventions directly against standard of care contact management. However, 68% of all Swazi child contacts under five who reportedly initiated TPT in Eswatini in 2019 were managed in our program. This occurred despite Vikela Ekhaya only being active in one region of the country and highlights the potential impact of community-based contact management and short-course child-friendly fixed dose regimens.

Adherence to TPT is essential to the prevention of TB disease in high-risk populations, including children under five years with household exposure to tuberculosis, especially in high-TB burden settings. This differentiated service strategy was successful in supporting child contacts and their caregivers from rural areas without sufficient access to care to initiate, remain adherent to, and eventually complete TPT in their homes. Poverty and urban residence were associated with missed doses and suggest that additional strategies may be needed in these populations. The association with self-reported missed doses and completion is important and speaks to the need for effective counseling based on self-reported adherence. Community based care also qualitatively increased access for female caregivers, and care teams were able to address concerns with the entire household. Perceptions around shorter TPT regimens were mixed, and indicates the need for patient education, particularly if different TPT regimens are being offered within the home. Overall, these results provide important evidence to inform the development and implementation of future large-scale preventive therapy programs in areas with high TB burden and bolster the identification of important subpopulations in need of further support.

## Supporting information

S1 Table Characteristics of children under 5 years initiating 3HR TPT. (DOCX)
