# Supplementary material for: Community-based tuberculosis contact management: Caregiver experience and factors promoting adherence to preventive therapy
Source: PLOS Glob Public Health. 2023 Jul 14;3(7):e0001920. doi: 10.1371/journal.pgph.0001920 (PMC10348572; doi:10.1371/journal.pgph.0001920)
Supplement: S1 Table — (DOCX) [file pgph.0001920.s001.docx]

S1 Table. Characteristics of children under 5 years initiating 3HR TPT

|  | Total |
| --- | --- |
|  | N=227 |
| Gender |  |
| Female | 117 (51.5%) |
| Male | 110 (48.5%) |
| Knowledge of HIV Status |  |
| Known | 213 (93.8%) |
| Unknown | 14 ( 6.2%) |
| Number of contacts per household | |
| 1-3 Contacts | 58 (25.6%) |
| 4-6 Contacts | 87 (38.3%) |
| 7-9 Contacts | 49 (21.6%) |
| 10+ Contacts | 33 (14.5%) |
| Travel time to facility |  |
| 0 to 30 minutes | 140 (61.7%) |
| 30 minutes to 1 hour | 66 (29.1%) |
| 1 to 2 hours | 14 (6.2%) |
| Missing | 7 ( 3.1%) |
| Monthly household income (USD) |  |
| 0 to 35 | 11 (4.8%) |
| 36-70 | 1. 11.9%) |
| > 70 | 182 (80.2%) |
| Missing | 7 (3.1%) |
| Household setting |  |
| Rural | 194 (85.5%) |
| Urban | 25 (11.0%) |
| Missing | 8 (3.5%) |
| Completed TPT on time | 216 (95.2%) |
| Patient Discontinued TPT | 1 (0.4%) |
| Clinician Discontinued TPT | 1 (0.4%) |
| Lost to follow up | 3 (1.3%) |
| Completed TPT late | 6 (2.6%) |
